# Hydrologic linkages drive spatial structuring of bacterial assemblages and functioning in alpine floodplains

**DOI:** 10.3389/fmicb.2015.01221

**Published:** 2015-11-03

**Authors:** Remo Freimann, Helmut Bürgmann, Stuart E. G. Findlay, Christopher T. Robinson

**Affiliations:** ^1^Department of Aquatic Ecology, Eawag: Swiss Federal Institute of Aquatic Science and TechnologyETH Zurich, Dübendorf, Switzerland; ^2^Institute of Integrative BiologyETH Zurich, Zürich, Switzerland; ^3^Department of Biology, Institute of Molecular Health Sciences, Epigenetic Regulation and Cell Identity ControlETH Zurich, Zürich, Switzerland; ^4^Department of Surface Waters – Research and Management, Eawag: Swiss Federal Institute of Aquatic Science and TechnologyETH Zurich, Kastanienbaum, Switzerland; ^5^Cary Institute of Ecosystem StudiesMillbrook, NY, USA

**Keywords:** hydrologic connectivity, eigenvector maps, bacterial community dynamics, ecosystem functions

## Abstract

Microbial community assembly and microbial functions are affected by a number of different but coupled drivers such as local habitat characteristics, dispersal rates, and species interactions. In groundwater systems, hydrological flow can introduce spatial structure and directional dependencies among these drivers. We examined the importance of hydrology in structuring bacterial communities and their function within two alpine floodplains during different hydrological states. Piezometers were installed in stream sediments and surrounding riparian zones to assess hydrological flows and also were used as incubation chambers to examine bacterial community structures and enzymatic functions along hydrological flow paths. Spatial eigenvector models in conjunction with models based on physico-chemical groundwater characteristics were used to evaluate the importance of hydrologically-driven processes influencing bacterial assemblages and their enzymatic activities. Our results suggest a strong influence (up to 40% explained variation) of hydrological connectivity on enzymatic activities. The effect of hydrology on bacterial community structure was considerably less strong, suggesting that assemblages demonstrate large functional plasticity/redundancy. Effect size varied between hydrological periods but flow-related mechanisms always had the most power in explaining both bacterial structure and functioning. Changes in hydrology should be considered in models predicting ecosystem functioning and integrated into ecosystem management strategies for floodplains.

## Introduction

Alpine floodplains consist of directional hydrologic networks that link different components of the floodplain landscape, including streams, lakes, riparian-, groundwater-, and hyporheic zones. These inherent linkages form a hierarchical and asymmetric dendritic web where connectivity depends strongly on the hydrologic state of the floodplain. Depending on the specific structure and hydrologic interactions within this network, different effects on physico-chemical and biological characteristics can be expected as hydrological connectivity changes (Kling et al., 2000). Floodplain morphology and hydrodynamics dictate the connectivity between hyporheic sediments and riparian areas. Mechanistically, this connectivity is determined in part by different water in- and ex-filtration rates as well as water residence times. These regional hydrological exchange properties influence physico-chemical processes (i.e., biotic and abiotic solute transformations during transport through the hydrological network) and thus generate habitat heterogeneity (Buffington and Tonina, 2009).

Bacteria play a key role in ecosystem functioning due to their ubiquity and integral role in metabolic processes. Bacterial community composition (BCC) can vary between habitats due to local environmental factors favoring better adapted and therefore competitive species (Logue and Lindström, 2010). Microbial functions (MF) also can vary among habitats as a direct consequence of species composition or additionally due to constraints by environmental factors. For instance, enzymatic activity, nutrient uptake, and microbial respiration in sediments can be regulated by local environmental conditions such as the quality of dissolved organic carbon, the water saturation state of sediments, and water temperature (Doering et al., 2011; Brockett et al., 2012).

The specific characteristics and degree of interaction among bacteria present in an ecosystem can influence community assembly and persistence (Lindström and Langenheder, 2011). Depending on phenotypic plasticity and the functional redundancy of a bacterial assemblage, changing environmental constraints may have different effects on community compositional trajectories and function. For example, Findlay et al. (2003) showed that hyporheic bacterial structure and metabolic functions changed due to altered dissolved organic matter composition, thus indicating little functional plasticity and/or low redundancy. In a similar study, nitrogen-amended hyporheic microbial communities changed their enzymatic activity patterns but not their community structure, indicating high functional plasticity (Findlay and Sinsabaugh, 2003). These findings suggest that species sorting depends on the specific environmental constraint in combination with the degree of functional plasticity and redundancy of specific traits within and between taxa in bacterial assemblages.

Regional hydrologic transport also directly affects species assemblages. Frequent dispersal from source habitats into recipient habitats influence the local species pool (Crump et al., 2007). Bacteria can be distributed actively or passively along hydrologic flow paths, thereby reaching downstream habitat patches (Cousin, 2009). The strength of mass flow in conjunction with the level of functional plasticity and redundancy of dispersed bacteria ultimately determines colonization success. For example, bacterial assembly in lakes with short retention times has been shown to be strongly influenced by mass effects, whereas lakes with long retention times were mainly characterized by local species-sorting mechanisms (Lindström and Bergström, 2004).

It is likely that a continuum of local (i.e., species sorting due to local environmental characteristics) and hydrologically-mediated regional processes (mass effects and changes in solute characteristics along flow paths) influence local BCC and MF. Importantly, temporal fluctuations in environmental factors within habitat patches not only constrain bacteria community assembly but also add an inertial component. For instance, bacteria that are early colonizers of a habitat patch may gain an advantage through the efficient use and “monopolization” of local resources (Urban and De Meester, 2009), and even MF can be altered by historical colonization events (Fukami and Morin, 2003). Ultimately, the relative strength and temporal variability of regional hydrologically-driven as well as local processes (both biological and physico-chemical) will influence BCC, MF, and beta diversity across habitat patches (Langenheder et al., 2011).

We conducted this study in a glaciated alpine catchment, assessing bacterial community assembly and functioning across hydrologically-connected habitat patches. The main objective was to gain insight into the mechanistic importance of hydrology in driving BCC and MF. We installed a nested set of piezometers in streams and in the adjacent riparian zone in two differently structured floodplains to assess community composition and potential enzyme activities of bacterial assemblages colonizing each piezometer. The floodplains had contrasting hydrological regimes due to the presence/absence of a proglacial lake, differences in riparian vegetation, and general landscape features, such as meanders, that influenced hydrological conditions. At one site, observations were repeated in different seasons to further assess contrasting hydrological regimes, thus expanding our range of inference. We hypothesized that hydrological connectivity, both underground and aboveground, influenced BCC and functioning. We expected to see shifting influences of regional vs. local mechanisms depending on different hydrological regimes. We used asymmetric eigenvector map (AEM) models to assess the importance of changes in regional linkages on bacterial assembly and functioning along flow paths. Symmetric eigenvector map models were built to emphasize non-directed locally driven processes. Additionally, a solute-dependent environmental model was used in partial redundancy analysis (RDA) to disentangle the environmental controls represented by each model component and to assess process patchiness.

## Materials and methods

Several abbreviations used throughout the manuscript are explained in Table 1.

**Table 1:** **Abbreviations and translations used in the manuscript and enzymes analyzed in this study, substrate used for assays and their biogeochemical functions**.

**Abbreviation**	**Translation**			
BCC	Bacterial Community Composition; assessed by ARISA (see below)			
MF	Microbial Function; assessed by eight enzymes (see below)			
RC	Roseg Creek			
SC	Simone Creek			
OTU	Operational Taxonomic Unit			
ARISA	Automated Ribosomal Intergenic Spacer Analysis			
MUF	Methylumbelliferone; used to assess enzymatic activities (see below)			
AEM	Asymmetric Eigenvector Map; models are used to investigate directional spatial processes			
MEM	Moran's Eigenvector Map; models are used to investigate non-directional spatial processes			
SDM	Solute Dependent Model; models are used in combination with AEM and MEM to assess spatial gradients or patchiness of solute characteristics			
IDW	Inverse Distance Weighting			
RDA	Redundancy Analysis			
NMDS	Non-metric Multidimensional Scaling			
VF	Vector Fitting			
**Enzyme (abbreviation)**	**Substrate analog**	**Acquiring element**	**Target**	**Function in ecosystem**
α-glucosidase (Alph)	4-MUF-α-D-glucoside	Carbon	α-1,4- and 1,6-glucosidic linkages	Starch degradation
β-glucosidase (Bet)	4-MUF-β-D-glucoside	Carbon	β-1,4-glucans	Cellulose degradation
β-xylosidase (Xyl)	4-MUF-β-D-xylopyranoide	Carbon	Xylose residues	Hemicellulose degradation
Esterase (Est)	4-MUF-acetate	Carbon	Small ester containing molecules	Glyceride hydrolization
N-acetyl-glucosaminidase (Nac)	4-MUF-N-acetyl-β-D-glucosaminide	Nitrogen	1,4-β-linkages of glucosamines	Chitin degradation
Leucine aminopeptidase (Leu)	L-leucine-7-amido-4-methylcoumarin	Nitrogen	Hydrophobic amino acids from N terminus	Peptide degradation
Endopeptidase (End)	4-MUF-4-guanidinobenzoate	Nitrogen	Peptide bonds	Peptide degradation
Phosphatase (Phos)	4-MUF-phosphate	Phosphorous	Phosphomono- and diester	Protein, Nucleotide degradation

### Site description and sampling

**Figure 1:**
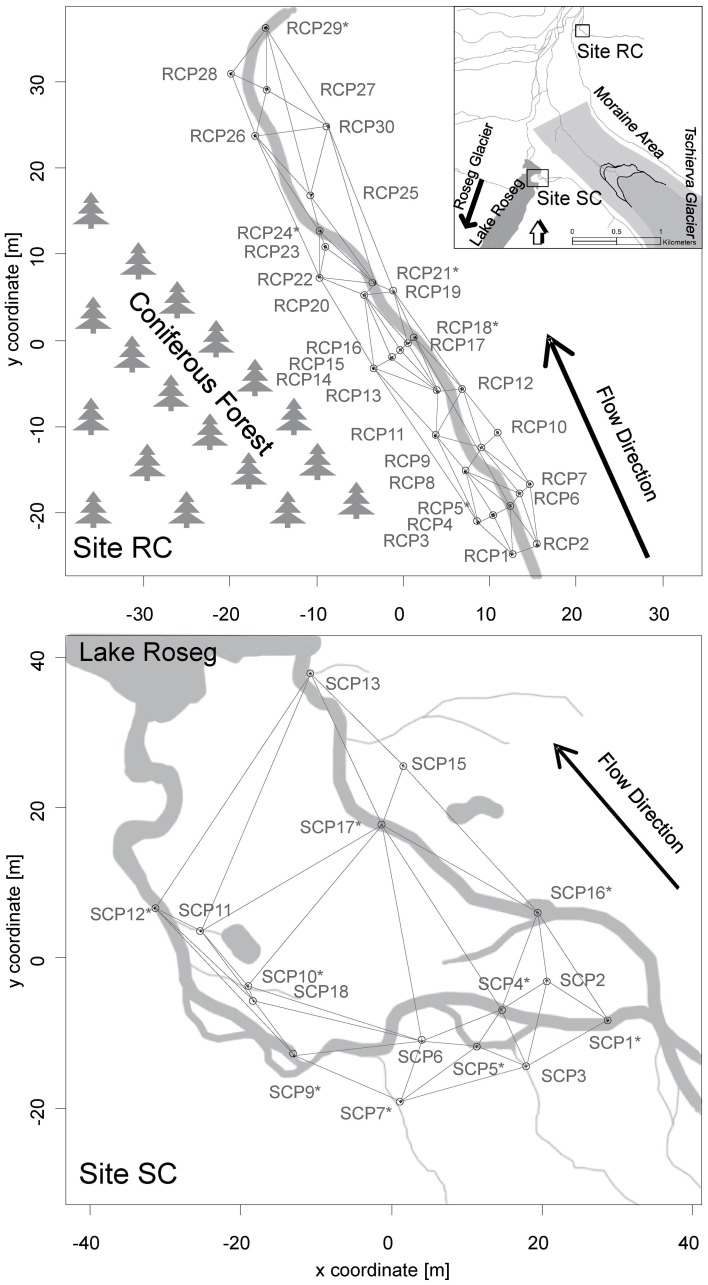
**Maps of the Val Roseg catchment and locations of the study floodplains with the upper panel showing site RC and the lower panel showing site SC**. Locations of piezometers in each floodplain relative to each stream, the direction of flow paths, and the spatial connectivity grid used for the AEM and MEM models are depicted. Asterisks depict piezometers located in-stream.

Study floodplains were associated with small tributary streams in the Val Roseg catchment, a glaciated alpine valley in the upper Engadin, Switzerland (Figure 1). Around 30% of the valley is glaciated (BAFU, 2010). We chose two floodplain sites that contrasted in regards to local tributaries, relative location within the valley, and their hydrological situation. One floodplain was associated with Roseg Creek (Site RC, N46°25"13′, E9°51"41′), a perennial groundwater-fed (krenal) tributary draining into the Roseg River. Elevation of this floodplain site is ca. 2044 m a.s.l. The floodplain is usually snow covered from November until May, and the riparian zone becomes saturated in spring due to nascent snowmelt. Toward summer, water saturation of the system decreases until it becomes wetted again from rain events and early snowfall in autumn. The riparian zone can be characterized as grassland with an adjacent coniferous forest (Figure 1).

The second floodplain was situated south of the moraine of the Tschierva glacier and harbors Simone Creek (site SC, N46°24"19′, E9°51"18′, Figure 1). The average elevation of this floodplain is around 2161 m a.s.l. In spring, the hydrology of the floodplain is mainly driven by snowmelt from snow packs on the side moraine and adjacent peaks. Minor flow during summer is from spring water and most floodplain channels run dry before autumn. In autumn, there is again increased water flow due to rain and snowfall events. The creek drains into Lake Roseg, a pro-glacial lake of the Roseg glacier complex. During hydrologic peak flows, the lower floodplain is wet and almost marsh-like. Soils near the lake are essentially water-logged most of the year. Riparian vegetation consists of shrubs (*Salix* spp.) and grass.

Thirty polyvinyl-chloride piezometers (86 cm long, 5 cm inner diameter, 5 mm pores) were installed in October 2008 at RC and 17 in July 2009 at SC, although only 15 were sampled at the latter location. The piezometers were fixed into in-stream sediments and riparian soils as depicted in Figure 1. Sterile, acid-washed glass beads (Braun B. Biotech, 1 mm diameter) were packed into individual 0.3 mm mesh nylon bags and placed at the bottom of each piezometer for colonization by bacteria. Beads were harvested at site RC in June after 4 weeks of incubation and in July after 3 weeks of incubation in 2010. Additionally, beads were harvested in October 2010 after 8 weeks of incubation at site RC and SC. The hydrological situation during incubations at site RC in June can be characterized as near bankfull stage, with melting snow patches saturating the surrounding riparian soil. In July site RC was in base flow stage and in October in increased base flow with rewetted riparian soil due to precipitation and snow fall. The hydrological situation at site SC during incubations can be characterized as increased base flow.

**Figure 2:**
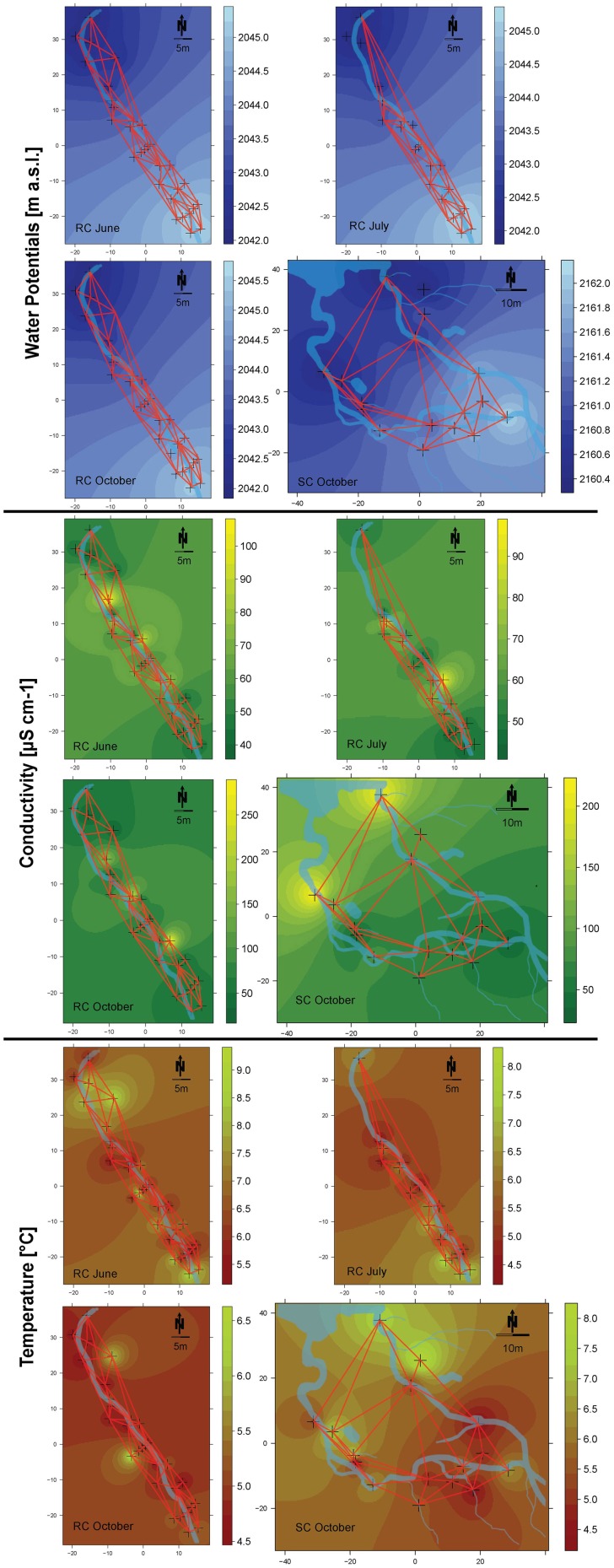
**Spatial maps of inverse distant weighting (IDW) inter- and extrapolations of water potentials (blue), conductivity (green), and temperature (red)**. Sampled locations are depicted as crosses on the maps. Refer to the respective color keys for inter- and extrapolated values of water potentials (depicted as m a.s.l.), conductivity (depicted as μS cm^−1^) and temperature (depicted as °C). The connectivity grid used for the different models and the streams are depicted on the maps.

Every incubation period started with newly installed bead bags. The different incubation times were due to scheduled sampling campaigns. Incubation time had no effect on the number of detected operational taxonomic units (OTUs), indicating no loss of represented bacterial diversity with shorter incubation time. Collected beads were rinsed with ultrapure water, placed in sterile centrifuge tubes and transported on ice to the laboratory within 12 h where they were stored at −20°C until further processing. Specific conductivity (μS cm^−1^ at 20°C) and temperature were measured within piezometers during bead harvesting with a conductivity meter (LF323; WTW) and visualized using IDW interpolation (Figure 2) (Cressie, 1993).

The geographical position of each piezometer was recorded with a geographic positioning system (Leica GPS 1200+) and a total station theodolite was used to determine the relative height level of each piezometer. The piezometers were placed to span a hydraulic head (m a.s.l.) difference of 3 m at site RC and 1.5 m at site SC. Single piezometers penetrated in-stream sediments and riparian soils approximately to a depth so that hydraulic heads within the piezometers were comparable. This depth relation guaranteed that local incubation conditions within piezometers were not different due to differences in water columns influencing, e.g., redox potential or pH. Hydraulic heads within each piezometer were measured on several dates in 2009 and 2010. Inverse Distance Weighting (IDW) interpolation of water potentials provided insight into potential water flow directions within each floodplain during the study period (Cressie, 1993; Figure 2).

Additionally, we qualitatively tested piezometer connectivity after the incubation experiments by injecting NaCl into several piezometers as a conservative tracer. A conductivity increase was observed over time between downstream located piezometers, thus confirming hydrologic connectivity.

### Bacterial community fingerprinting

Bacterial community structure was assessed by automated ribosomal intergenic spacer analysis (ARISA). The PowerSoil DNA isolation Kit (MoBio, Carlsbad) was used to extract DNA from the incubated glass beads (~1 g, *n* = 2) following manufacturer's instructions. DNA was amplified using the fluorescein (6-FAM) labeled universal forward primer 1406f-6FAM (16S rRNA gene, 5′-FAM-TGYACACACCGCCCGT-3′, Y = T,C) and the bacteria specific reverse primer 23Sr (5′-GGGTTBCCCCATTCRG-3′, B = G,T,C, R = G,A) (Yannarell et al., 2003). PCR and ARISA fragment analysis was performed as described in Bürgmann et al. (2011). Binning of peaks was done with automatic and interactive binning scripts in R (Ramette, 2009) and relative fluorescence intensity of binned peaks between 200 and 1400 bp were averaged and used for subsequent statistical analysis.

### Enzyme assays

Eight enzymes were tested for their potential activity using Methylumbelliferone (MUF)-labeled substrate analogs (Sigma-Aldrich CO, St Louis, MO, USA, see Table 1). Enzymes that degrade polysaccharides were tested using 4-MUF-α-D-glucoside for Alpha glucosidase (Alph), 4-MUF-β-D-glucoside for Beta glucosidase (Bet) and 4-MUF-β-D-xylopyranoide for β-xylosidase (Xyl). 4-MUF-N-acetyl-β-D-glucosaminide was used to assess hydrolysis by N-acetyl-glucosaminidase (Nac) (Sinsabaugh et al., 2008). Esterase (Est) activity was measured with 4-MUF-acetate (Arpigny and Jaeger, 1999). Leucine aminopeptidase (Leu) and endopeptidase (Epep) activity were measured using L-leucine-7-amido-4-methylcoumarin and 4-MUF-4-guanadinobenzoate, respectively (Vihinen and Mäntsälä, 1989; Makoi and Ndakidemi, 2008). 4-MUF-phosphate was used to assess phosphatase (Phos) activity.

Approximately 10 g of frozen glass beads were thawed on ice and subsequently transferred to a sterile centrifuge tube containing 5 ml ultrapure water. Enzymes were released by vortexing the bead-water mix for 2 min. Three replicates of supernatant per resolved enzyme solution were transferred into a 96 well microplate and substrate stock solution was added to a final concentration of 400 μM (Findlay et al., 2001). The remaining beads were washed with ultrapure water and dried at 60°C for 48 h to assess the exact weight of the beads to calculate the initial bead surface area. Enzyme potential activity rates were measured by collecting fluorimetric data 10 min and 1, 2, 3, 8, 20, and 24 h after adding the substrate using a microplate reader (Teacan Infinite 200, Switzerland) and excitation/emission wavelengths of 365/445 nm. The microplates were stored on a plate shaker at 15°C between measurements. Fluorescence values were corrected for quenching by adding known quantities of MUF solution to the samples and comparing them to the fluorescence increase when MUF was added to buffer (Findlay et al., 2001). Reaction rates were calculated using the slope of the linear part of the fluorescence reaction curve. Potential enzyme activities were standardized to nmol substrate m^−2^ bead area h^−1^. It is possible that part of the measured bacterial enzymes might originate from fungal biomass attached to the beads. However, the contribution of fungal enzymes to the biofilm total enzyme activity is likely negligible as fungal biomass was found to be barely detectable on submerged glass plates (Sinsabaugh et al., 1991).

### Data analysis

**Figure 3:**
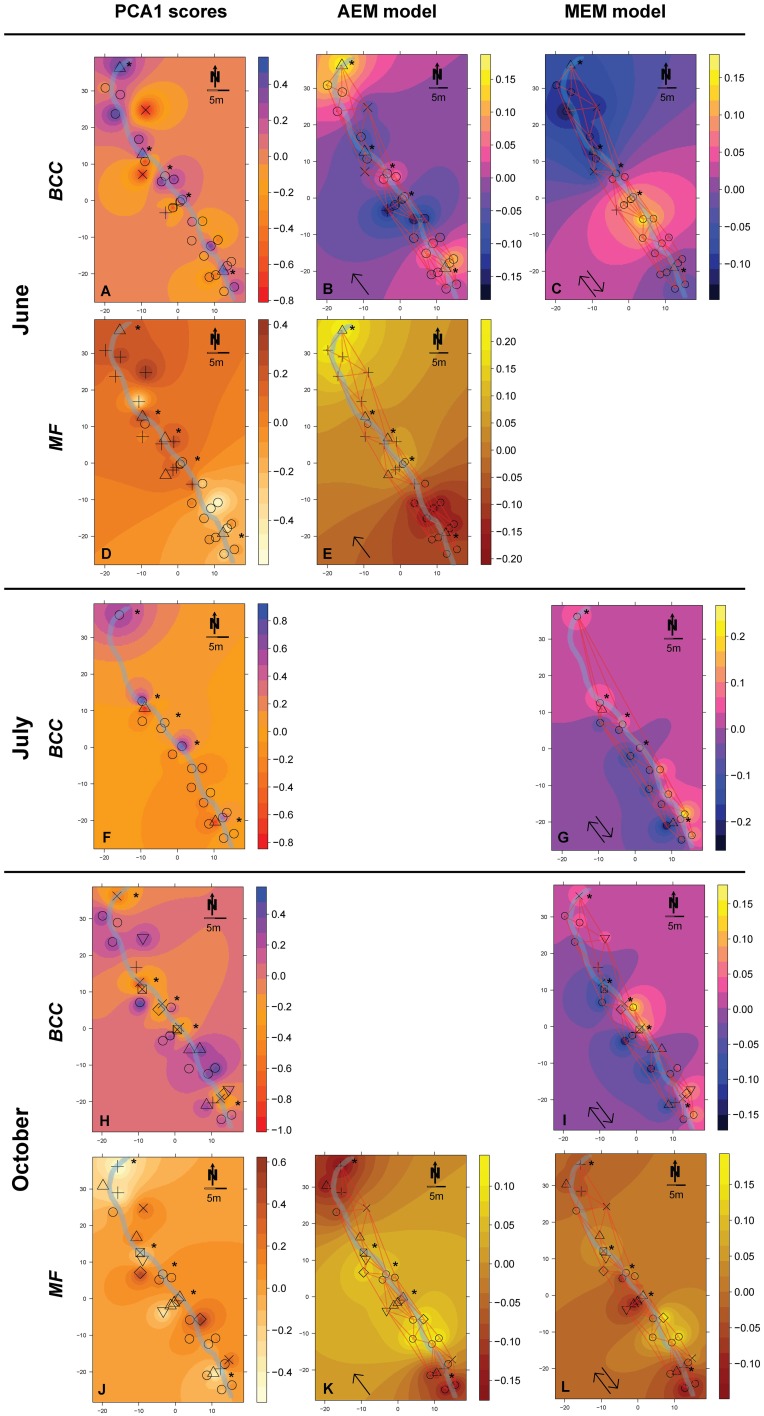
**Spatial maps of IDW inter- and extrapolations of PCA1 scores and first RDA axis values constrained by spatial explanatory variables from the significant AEM and MEM models for BCC (pink-yellow color scheme) and MF (orange color scheme) at site RC**. The piezometer locations and the used connectivity grid are depicted. Symbols represent the relative affiliation of MF or BCC derived from the cluster analysis; i.e., same symbols indicate a high similarity according to this parameter. Asterisks depict piezometers located in-stream. Arrows depict if the interpolation is based on the AEM model (single arrow) or from the MEM model (double arrow). **(A–C)**, BCC in June; **(D,E)**, MF in June; **(F,G)**, BCC in July; **(H,I)**, BCC in October; **(J–L)**, MF in October. Non-significant models are not shown (empty panels).

**Figure 4:**
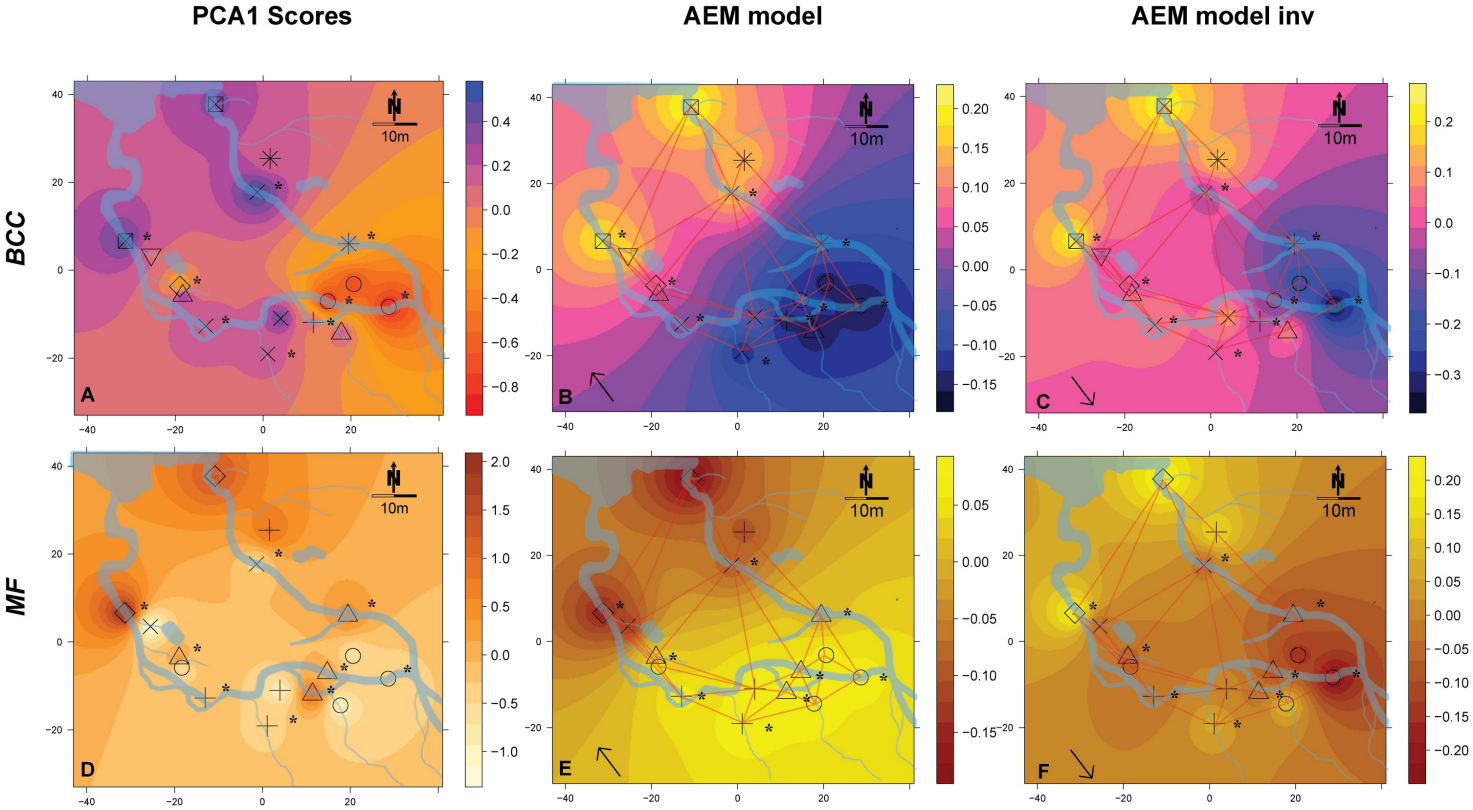
**Spatial maps of IDW inter- and extrapolations of PCA1 scores and first RDA axis values constrained by spatial explanatory variables from the significant AEM models for BCC (pink-yellow color scheme) and MF (orange color scheme) at site SC**. Piezometer locations and the used connectivity grid are depicted. Symbols represent the relative affiliation of MF or BCC derived from the cluster analysis; i.e., same symbols indicate a high similarity of samples for this parameter. Asterisks depict piezometers located in-stream. Arrows depict that the interpolations are based on AEM models (single arrow). **(A–C)**, BCC; **(D–F)**, MF. Non-significant models are not shown.

Hierarchical clustering of ARISA community fingerprinting data (structure, BCC) and enzymatic activity data (function, MF) was performed for each of the different incubation periods by applying the Unweighted Pair Group Method with Arithmetic Mean method to the Bray–Curtis dissimilarity matrix of each data type. An optimal number of clusters was determined using the Rousseeuw quality index (Rousseeuw, 1987). Additionally, Mantel correlations between the original distance matrix and binary matrices from groups produced by cutting the dendrogram at different height levels were computed. The highest correlation equals the best clustering. Where the optimal number of clusters was not the same for both methods, silhouette widths were calculated for the range in the number of clusters and the largest number of clusters was chosen where no negative silhouette width (i.e., potential mismatch of objects in the clusters) occurred (Borcard et al., 2011). The assessed clusters for BCC and MF are depicted as different symbols in Figures 3, 4.

Permutational multivariate analysis of variance (PERMANOVA) was used to test the influence of the two streams (RC, SC), piezometer location (in-stream, riparian zone) and season on BCC and MF (Anderson, 2001). Different models were built: A total model using all samples to test the effect of floodplain system and location, a model for the SC system testing the influence of piezometer location, and a model for the RC system to test the influence of piezometer location and season. In addition, a pairwise comparison of the two floodplain systems was performed (Table 2). Models were run on Wisconsin transformed ARISA community fingerprinting data and raw enzyme data, respectively.

**Table 2:** **PERMANOVA of BCC and MF**.

**Variable**	**Systems**	**Factor**		**r^2^**	**F**	**P**
Structure	RC and SC	System (Sy)		0.03	3.25	< 0.001
		Location (L)		0.05	4.99	< 0.001
		Sy × L		0.03	2.82	< 0.001
	RC	L		0.08	6.68	< 0.001
		Season (S)		0.03	1.40	< 0.025
		L × S		0.02	0.69	0.970
	SC	L		0.08	1.21	0.174
		**Pairwise Comparison**		**r^2^**	**F**	**P**
		RC RI	SC RI	0.02	1.13	0.259
		RC RI	SC IS	0.04	3.09	< 0.001
		RC IS	SC RI	0.16	3.77	< 0.001
		RC IS	SC IS	0.22	6.29	< 0.001
**Variable**	**Systems**	**Factor**		**r^2^**	**F**	**P**
Function	RC and SC	System (Sy)		0.08	9.48	< 0.001
		Location (L)		0.08	9.44	< 0.001
		Sy × L		0.03	3.59	< 0.01
	RC	L		0.13	12.74	< 0.001
		S		0.10	4.77	< 0.001
		L × S		0.02	0.94	0.451
	SC	L		0.07	1.13	0.332
		**Pairwise Comparison**		**r^2^**	**F**	**P**
		RC RI	SC RI	0.04	2.51	0.053
		RC RI	SC IS	0.08	6.08	< 0.001
		RC IS	SC RI	0.32	9.57	< 0.01
		RC IS	SC IS	0.40	14.38	< 0.001

*Models are built for between systems comparison, comparison of Location, Season and Location × Season at system RC and for Location at system SC. A pairwise comparison of in-stream (IS) and riparian (RI) locations between the systems is also given*.

#### Spatial models

Spatial dynamic scenarios of BCC and MF were evaluated by means of different spatial eigen function models in conjunction with solute dependent models (SDM).

#### Directional hydrological driven processes

We used AEM models developed by Blanchet et al. (2008b, 2011). These models were designed to investigate directional spatial processes. Such processes are likely to occur in floodplains along hydrologic connectivity networks in the direction of water flow with upstream sites influencing downstream sites. Thus, AEM models can shed light on the extent to which hydrologic flow influences bacterial community assembly and microbial function (i.e., enzymatic activity). Mechanistically, these directed processes are thought to be linked to passive bacterial dispersal and the continuous spatial structuring of flow dependent environmental variables such as physico-chemical gradients or consecutively processed nutrients. Separate models were produced for each sampling period and the two floodplains. Connectivity matrices (sites-by-edges matrices) were built in concordance with measured water potential and with the qualitative tracing patterns of NaCl injections (see Figures 1, 2). They include information about links between piezometers and water flow direction (Blanchet et al., 2011; Borcard et al., 2011). Additionally, weighting factor matrices were introduced based on the function of distance: *f* (*d*_*ij*_) = 1 - (*d*_*ij*_/max(*d*_*ij*_)), where *d*_*ij*_ represents the individual distances between linked piezometers (Borcard et al., 2011). The weights reflect a higher likelihood of transport connectivity between linked piezometers. The connectivity matrices were combined with the weighting factors by the Hadamard product of matrices. Spatial eigenfunctions were calculated by principal component analysis (PCA) of the weighted connectivity matrix (Blanchet et al., 2008b).

For site SC, we also developed an inverse AEM model that has inverted directionality (i.e., from lake toward upstream sites). We chose to make these models for site SC as a pro-glacial lake was situated at the downstream site of the floodplain. Previous phosphorous uptake measurements within this floodplain indicated that lake water can penetrate the floodplain via porous alluvium and thus may introduce an environmental gradient opposite to the above ground flow direction (data not shown).

#### Non-directional processes

Moran's Eigenvector Map (MEM) modeling was used to assess the influence of non-directed processes between piezometers that are in spatial proximity (Borcard and Legendre, 2002; Borcard et al., 2004; Dray et al., 2006). Non-directed spatial patterns influencing BCC and MF can be generated, e.g., by local dispersal due to rain events or habitat patch characteristics affected by spatial proximity (i.e., due to elevation changes or local sediment characteristics). We expect these mechanisms to be of higher importance when directed water flow is minimal and water saturation is low. We used the same connectivity matrices and weighting function as in AEM models, except for the lack of directionality information of the site by edge matrices. Principal coordinate analysis of the weighted Euclidian distance matrix was used to derive the spatial eigenfunctions (Borcard et al., 2011).

AEM and MEM eigenfunctions with positive eigenvalues were tested for positive spatial correlation based on Moran's I (Cliff and Ord, 1981). Significant positively correlated eigenfunctions were then used as descriptors in RDA on structures (BCC) and functions (MF) as response variables. Forward selection of eigenfunctions was performed to ensure selection of parsimonious models (Blanchet et al., 2008a). Significance of constraints (i.e., eigenfunctions) and the canonical axes were tested by permutation tests (999 permutations, “marginal” testing method, Legendre et al., 2011). Fitted values of the significant first RDA axis were then plotted on the piezometer site maps using IDW interpolation to visualize spatial structuring within the floodplains (Figures 3, 4). Adjusted R^2^ (R${}_{a}^{2}$) was calculated to assess the percentage of variation explained by the models (Peres-Neto et al., 2006). The response variables used in AEM and MEM were Hellinger transformed prior to analysis and detrended for MEM modeling when necessary (Borcard and Legendre, 2002; Borcard et al., 2011).

#### Solute dependent processes

To evaluate the influence of solute related mechanisms in determining BCC and MF, we constructed RDA models investigating the correlation between BCC and MF and local solute characteristics (i.e., conductivity and temperature). Solute characteristics may form a continuous gradient with local patchiness depending on several mechanisms. At the presented spatial scale, solutes are most likely affected by strength in vertical (stream) water flow, sediment/soil characteristics, and floodplain morphology (Salehin et al., 2004). For example, these factors can lead to up- and downwelling events that influence local solute characteristics (i.e., Storey et al., 2004) and ultimately form physical habitat templates within floodplains (Poff and Ward, 1990; Dole-Olivier et al., 1994).

**Figure 5:**
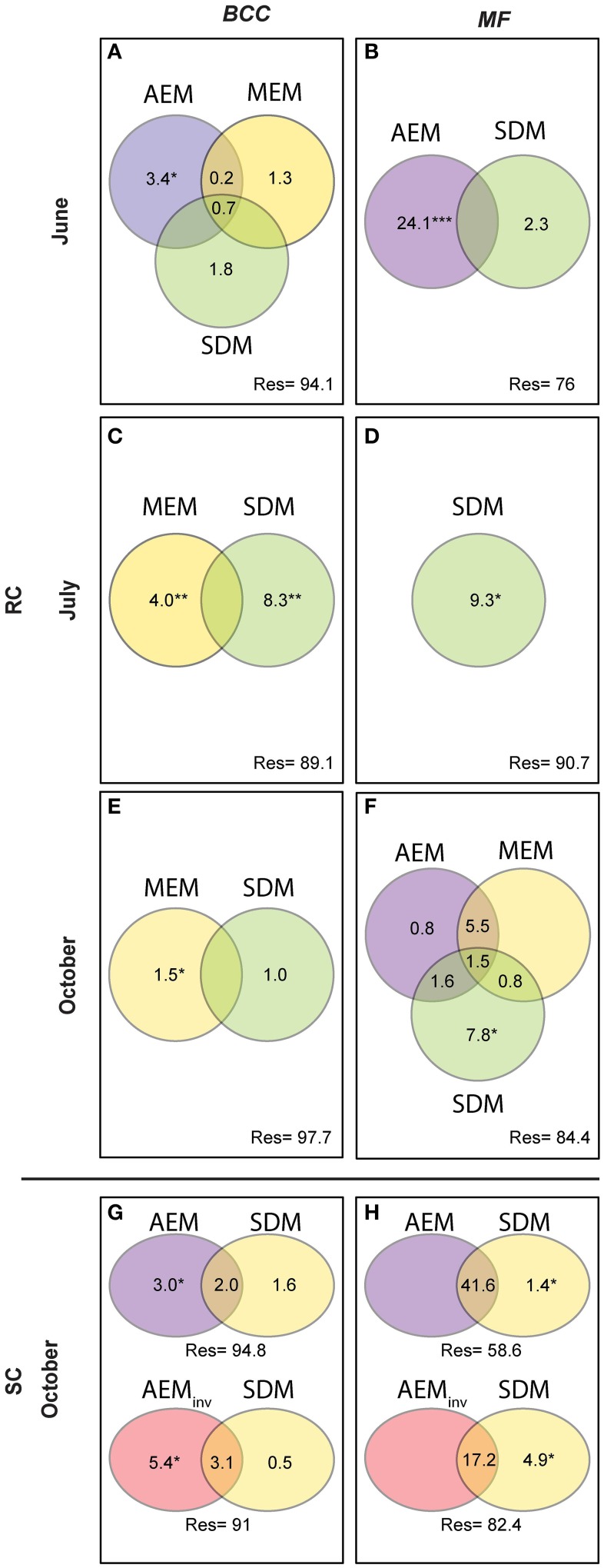
**Variation partitioning analysis performed for BCC and MF on significant spatial (AEM and MEM) and solute dependent models (SDM)**. AEM and its shared fractions with MEM models catch spatially-directed processes such as passive downstream transport of bacteria or consecutively-processed nutrients structuring BCC and MF. Solute structuring (i.e., physico-chemical groundwater characteristics) actively driven by water flow (downstream) influencing BCC and MF is captured by the shared fraction of AEM and SDM. Patchy/local solute structuring (i.e., during low water flow or strong up-downwelling events) driving BCC and MF is captured by SDM and the shared SDM and MEM components. MEM fractions relate to point-source mechanism (i.e., local dispersal due to rain events or local sediment characteristics). Upper panel represents the RC floodplain during the different seasons. Lower panel represents the SC floodplain. Values are equal to % explained variance of BCC or MF with respect to specific model variables or their combined effects. Negative values are not shown. ^*^P ≤ 0.05, ^**^P ≤ 0.01, ^***^P ≤ 0.001. **(A,B)**, BCC and MF at site RC in June; **(C,D)**, BCC and MF at site RC in July; **(E,F)**, BCC and MF at site RC in October; **(G,H)**, BCC and MF at site SC in October.

**Figure 6:**
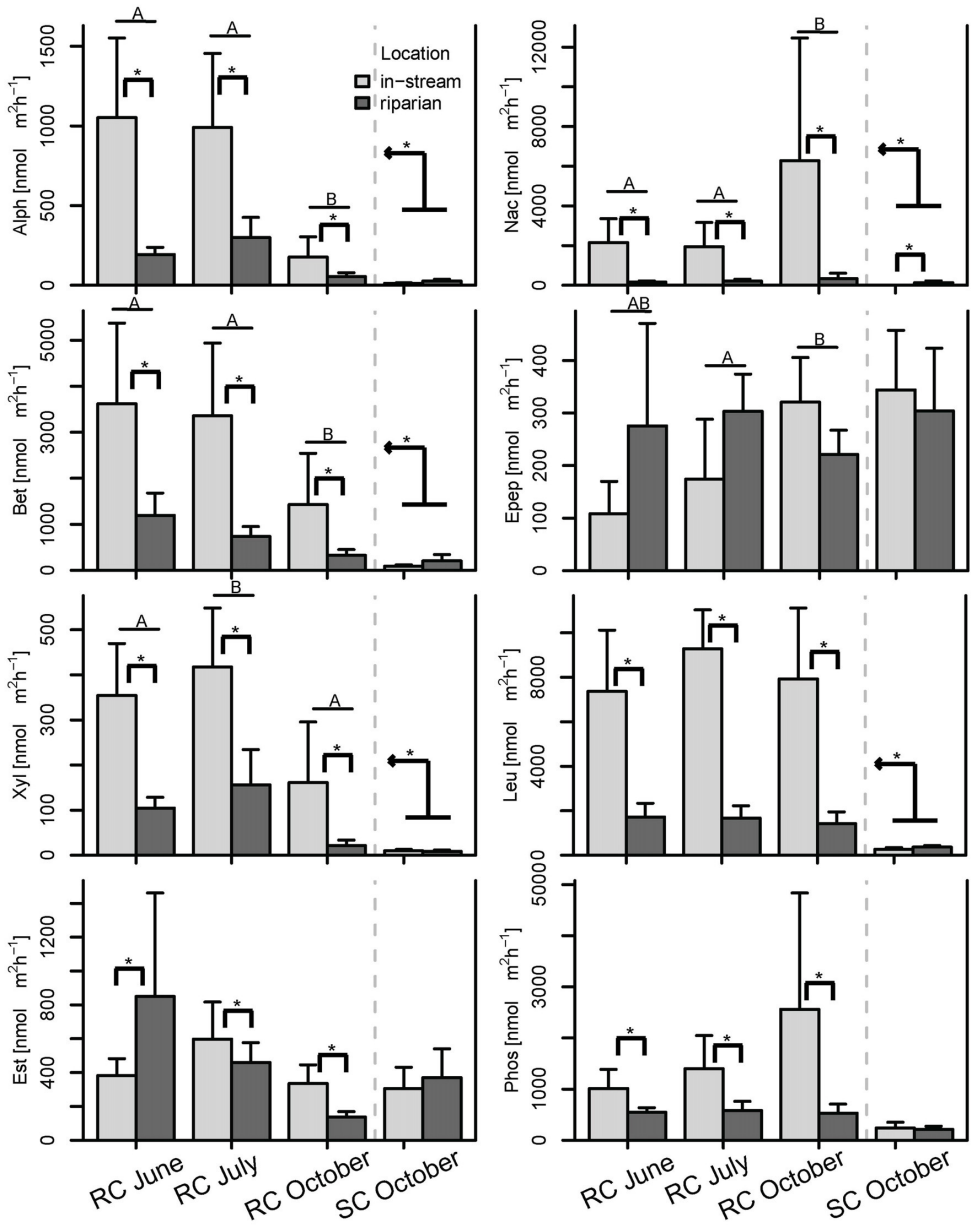
**Bar plot of the enzymatic activities split by season and location**. *Post-hoc* test results are displayed as follows: Bars with different letters show significant differences between seasons, brackets with asterisks depict significant differences between concurrently sampled locations and the kinked arrow with asterisk shows overall differences between the two systems across all samples (*p* < 0.05). Whiskers are standard errors.

We performed variation partitioning between selected AEM, MEM, and SDM using partial RDA (see **Figure 6**, Blanchet et al., 2011; Bertolo et al., 2012) to evaluate the performance of the spatial models (AEM and MEM) and gain insight into underlying mechanisms related to solute dependent processes in structuring BCC and MF. Unique fractions of the variation partitioning analysis were tested by permutation tests with 999 randomizations (Peres-Neto et al., 2006). Solute dependent variables were normalized by log transformation prior to analysis. Negative fractions are not shown in Figure 5.

Further, PCAs from Hellinger transformed BCC and MF were produced and site scores of principal component axes were used for IDW interpolation to visualize spatial variation of the original data sets (see Figures 3, 4 and Figure S1). Lastly, analysis of variance (ANOVA, Type III SS) tested for differences in the number of OTUs and enzyme activities between floodplain systems. Additional ANOVA models were built for single enzymes to assess differences between floodplains, the piezometer locations within the SC system, and the influence of piezometer and sampling date within the RC system. If the interactions or single factors were significant, a Tukey's *post-hoc* test was performed and included in Figure 6. All analyses were performed using R statistical software (R Development Core Team, 2015).

## Results

### Spatial and temporal patterns of BCC and MF: Roseg creek

*Hydrologically Active Period: June*- Community structure data revealed a separation of in-stream (IS) and riparian zone (RI) BCC in June (see dispersion ellipses Figure S2). The AEM model explained 3.2% of the variation and showed a spatial structuring (i.e., the color patterns of the AEM map) of BCC that was divided into upstream (lower right corner)/downstream (upper left corner) and midstream (i.e., water flowing in direction of depicted arrow) (Figure 3B). Non-directional MEM models explained 1.8% of bacteria assemblage structure (Figure 3C). Temperature and conductivity showed no significant association with bacterial community structure. Temperature and conductivity patterns were generally not congruent with PCA scores of BCC (i.e., compare Figure 2 with Figures S1A,B). Variation partitioning revealed that water flow best explained BCC patterns (Figure 5A).

In this June period, MF showed a clear separation between IS and RI locations and revealed an additional separation between riparian upstream and downstream piezometers (Figure 3D, see dispersion ellipses in Figure S2D). Some 21.8% of the functional variation could be explained by the directional AEM model (Figure 3E). Conductivity and temperature were not associated significantly with the MF pattern and variation partitioning showed no shared fraction of AEM and SDM models (Figure 5B).

*Hydrologically Inactive Period: July*- BCC showed no distinct clustering during this period expect for subtle differences between in-stream and riparian sites (Figure 3F, Figure S2C). No significant AEM model could be built for structure or function in July. The MEM model for bacteria structure explained a small proportion (2.6%) of the total variation (Figure 3G) and there was no significant MEM model for MF in July. The SDM model explained 8.3% of variation in BCC in the variation partitioning with no shared fraction with the MEM model (Figure 5C). Some 9.3% of the variation of the MF was explained by the SDM model (Figure 5D). MF was generally equalized over the whole floodplain according to the first PCA axis scores, which explained 64.6% of the MF variation (Figure S1G). Scores of the second axis of the MF PCA (explaining 13.5% of the variation, Figure S1H) showed a similar pattern as the IDW interpolation of temperature and conductivity (Figure 2).

*Re-establishing Hydrological Linkages: October*- In October, there appeared to be increasing structural and functional beta-diversity in the floodplain as shown by a larger number of clusters and more distinct structuring according to the PCA scores distribution (Figure 3H, Figures S1I,J). In-stream BCC differed from the riparian BCC (Figure S2E). No significant AEM model could be built for BCC. The MEM model explained 1.3% of the bacteria structure (Figure 3I). MF showed more spatial structuring than in July (Figure 3J). The AEM model explained 9.3% of MF and mostly covered an upstream/downstream vs. mid-stream structuring of enzymatic activities (Figure 3K). The MEM model explained 5.3% of MF and reflected the finer spatial structuring as seen in the PCA scores (Figure 3L, Figures S1K,L). Variation partitioning revealed that MF was mainly explained by directional processes (6.3%), directional processes linked to SDM model components (3.1%) and to SDM components not linked to directed spatial structuring (8.4%, Figure 5F).

### General findings for Roseg creek

BCC and MF patterns differed between in-stream and riparian zone piezometer locations at site RC and also showed a shift between the different hydrological periods (Table 2, also see dispersion ellipses in Figure S2). In-stream communities had higher total enzymatic activities than riparian communities in the RC system [ANOVA: *F*_(1, 72)_ = 24.73, *P* < 0.001]. This pattern was true for seven out of eight measured enzymes (Figure 6).

### Spatial patterns of BCC and MF: Simone Creek

There was a change in BCC toward Lake Roseg as seen with the annotated clusters and the PCA scores IDW interpolation (Figure 4A). This pattern could be modeled with the AEM, which explained 5% of the variation in bacterial composition (Figure 4B). The inverse AEM explained 8.4% of the BCC structuring (Figure 4C). MF showed the same patterns as for bacterial structure: a gradual cluster and BCC structuring toward Lake Roseg (Figure 4D). The directional AEM model explained 40% of the variation in enzyme activity (Figure 4E). The inverse AEM model explained 12.7% of the enzyme activity patterns (Figure 4F). Variation partitioning showed that AEM and inverse AEM models best explained the variation of BCC and MF (Figures 5G,H). SDM model components that were not structured in either floodplain direction showed an influence on MF. SDM model components showed a large shared fraction with AEM for both, BCC and MF. No significant MEM models could be built for BCC or MF for Simone Creek.

### General findings for Simone Creek

No difference between in-stream and riparian zone was found at site SC concerning BCC and MF patterns and total enzyme activity (Table 1, Figure 6, dispersion ellipses Figures S2G,H).

## Discussion

Hydrological connectivity has been shown to be an important factor in forming communities along riverine dendritic networks (Besemer et al., 2013; Liu et al., 2013). Nonetheless, many studies ignore the fact that riverine ecosystems are composed of different hydrologically-linked components, not taking into account interactions with riparian-, groundwater-, or hyporheic zones. Alpine floodplains represent an excellent possibility to study the effects of such interactions on the mechanisms influencing bacterial communities as well as ecosystem functions since they consist of hierarchically-structured and hydrologically-interconnected habitat templates. Floodplain compartments experience annual changes in their hydrologic state and thus differ in strength of connectivity throughout the year. The degree of connectivity is mainly determined by floodplain morphology and water input.

**Figure 7:**
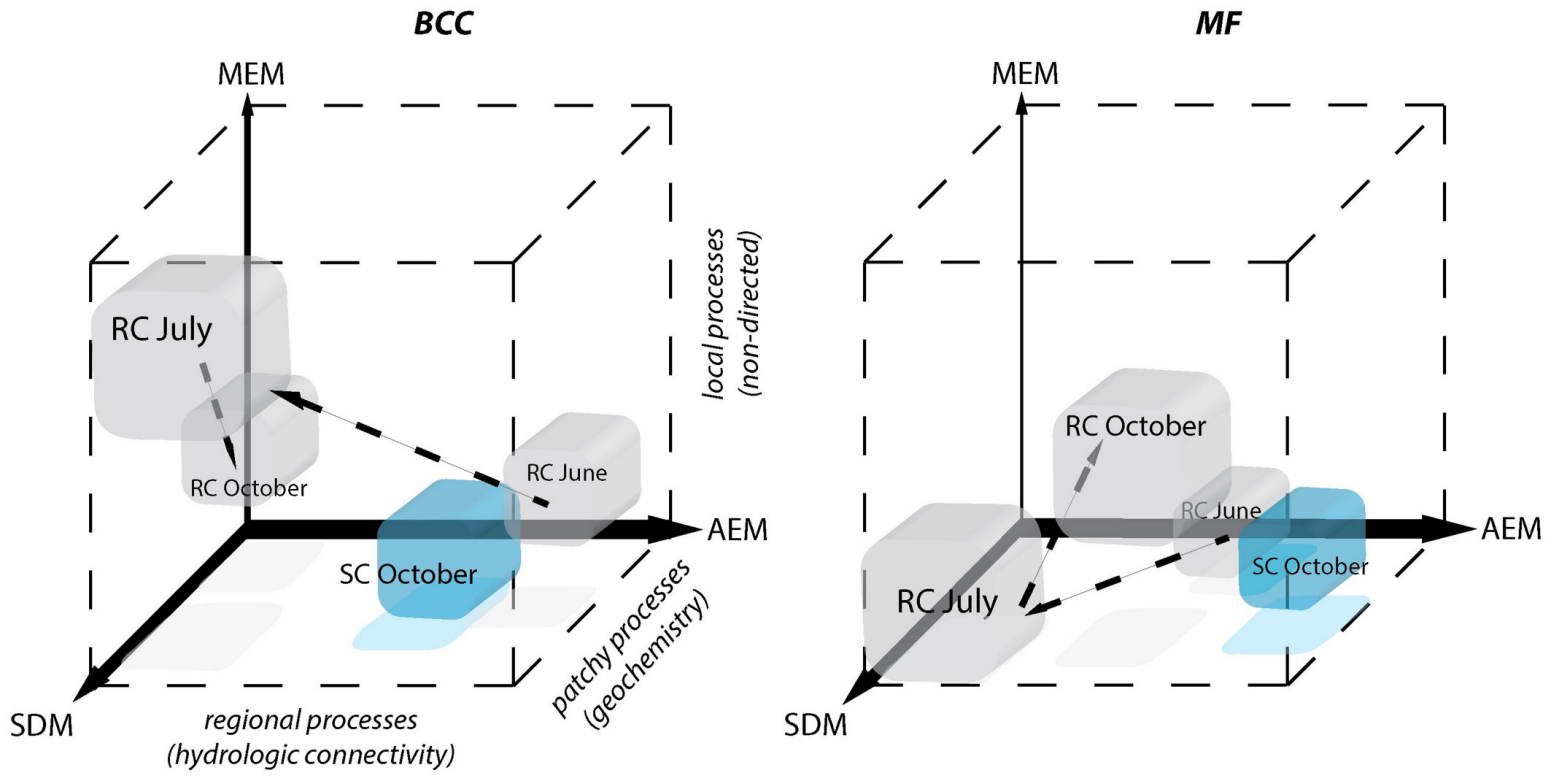
**Schematic depiction of the relative contributions of processes driving BCC and MF during different hydrologic periods**. Positions of the cubes indicate the relative importance of each of the three models for the structuring of BCC and MF. AEM describes regional hydrologic drivers (downstream), MEM describes local connectivity related drivers that are not directed and SDM describes patchy solute characteristics. Patchy solute characteristics become more spatially structured (i.e., locally or regionally) when MEM or AEM model components gain importance simultaneously with SDM. The power of the different model components compared to one another is indicated by the width of the respective axes. Gray cubes correspond to the RC floodplain and successive changes during different hydrological periods are indicated by arrows. SC floodplain is depicted as a blue cube.

The use of eigenvector models revealed that regional hydrological connectivity locally influences BCC and MF. Different mechanisms in forming assemblages and ecosystem functions are favored depending on the relative strength of directional water flow and interconnectivity of different floodplain compartments. The relative contributions of different processes are schematically summarized in Figure 7 and addressed below.

### Regional processes (AEM)

Pronounced regional hydrologic connectivity through the entire floodplain area can establish a directional change in BCC and BF along the flow path (i.e., AEM component, Figure 7). Mechanistically, these directional changes in BCC can be linked to bacterial transport (i.e., mass effect) and/or gradual changes in solute characteristics along the flow path introducing spatially structured habitat patches. The AEM model components (and its shared fractions with SDM model components) were used to analyze these regional mechanisms in the present study. Floodplain morphology and hydrodynamics (i.e., total water input or the relative contribution of stream water vs. riparian water flow) were shown to determine the strength in linkage within and between floodplain compartments in June at site RC and October at site SC. For example, regional hydrologic drivers can act compartment-specific when groundwater flow is in parallel to channel flow. This hydrologic situation is characteristic for the RC floodplain during high water input in June, thus allowing for separated structuring of in-stream vs. riparian BCC. As groundwater flow is directed across meandering floodplain streams and multiple small ephemeral tributaries, more exchange of in-stream groundwater, in-stream water and riparian water can be expected (i.e., at site SC) and BCC becomes more similar along the flow path across all compartments. Previous studies have shown that bacteria can be washed from soils and be transported into streams (Cousin, 2009). Such hydrologically driven inputs can even influence larger ecosystems such as lakes, thus potentially driving the lake community to resemble the in-stream community (i.e., a mass effect) (Lindström and Bergström, 2004; Crump et al., 2007).

MF, in comparison to BCC, was more strongly affected by hydrologic connectivity at times of active hydraulic transport (i.e., RC in June and RC and SC in October), with compartment separations according to floodplain morphology (see also Figure S2). A longitudinal change in enzymatic activity may be driven by a gradual shift in nutrient and organic matter resources to more recalcitrant forms along the flow path (Sobek et al., 2003; Ayuso et al., 2011). For instance, *Epep* and *Est* showed more activity at locations further downstream compared to enzymes that degrade more bioavailable compounds at upstream locations (Figure S2). In alpine floodplains, organic matter input (quantity, quality, and source) can fluctuate widely among seasons (Hood et al., 2005). Most in-stream primary production occurs during summer. Leaching of organic matter from riparian soils and vegetation occurs during the hydrologically active seasons in autumn and spring as a consequence of riparian and groundwater flow (Boyer et al., 2000; Sawyer et al., 2014). In spring, there is additional organic matter and nutrient input from snowmelt runoff. The flow-paths determine the processing and consumption of organic matter and nutrients within and across compartments and thus define the spatial structure of BF within floodplains. This was mirrored by the different longitudinal structuring of BF in our study floodplains during hydrologically active periods. For example, the between-compartment exchange was more apparent at site SC, which is explained by the meandering waterways that led to a clear longitudinal structuring of BF across compartments (thus the large explained variation by AEM model component). In comparison, compartments at site RC were more separated due to the alignment of ground and surface water flows.

In addition to affecting BF, changes in nutrients along the flow path can also introduce a spatially-structured species sorting effect that locally influences BCC. Accordingly, we observed BCC to change in congruence with bacterial functions at site SC (see cluster affiliation and idw structures of BCC vs. BF in Figure 4).

### Patchy processes (SDM)

Solute characteristics that are not linked to directional water flow can work as patchy environmental constraints driving BCC and MF (i.e., SDM component, Figure 7). This component became important during the drier summer period at site RC when water saturation within the floodplain decreases. The riparian zone was relatively uniform in BCC and MF, likely due to a lack of hydrologically-maintained gradients in water chemistry that could have affected BCC and MF. Nevertheless, stagnation of water led to a patchy solute structuring (i.e., large range in conductivity). Species sorting mechanisms in conjunction with constrained MF due to solute characteristics were likely important in forming BCC within the RC floodplain in June. Local environmental filtering also has been shown to become more important in riverine ecosystems during decreased discharge (Liu et al., 2013). Alternatively, SDM processes can be linked to up- or downwelling events within a floodplain. This mechanism is likely to influence BF at site SC (see below).

### Non-directed local processes (MEM)

Non-directed local processes can be linked to, e.g., non-directed dispersal due to rain events, input of organic matter due to local litter decomposition or local sediment characteristics (i.e., MEM component, Figure 7). In our study, these mechanisms least explained BCC and BF structuring. The MEM component revealed a BCC separation between the two riparian zones separated by the stream during the summer dry period (Figure 3G).

### Process continuum

Regional, local, and patchy processes belong to a mechanistic continuum. These process components can operate independently or synergistically. The leverage of these components differs in the context of the continuum and the dependent variable. Furthermore, they can be “hijacked” by physically more powerful components (i.e., shared fractions of model components, Figure 5). In our study systems, the explanatory power of the process components was dominated by hydrologically-driven regional processes followed by patchy and local processes for both BCC and BF (see arrow thickness Figure 7). Such a process continuum could be observed at site SC. During phosphorous uptake experiments within the SC floodplain, we detected unexpected patterns in water phosphorous concentrations (data not shown). Phosphorous concentrations were higher toward the pro-glacial Lake Roseg or showed peaks in the middle of the floodplain. Actually, the inverse AEM model (AEM_inv_) explained up to 8.4% of BCC variation with a larger shared fraction of the SDM. It seems that lake water affects solute characteristics of the floodplain via the alluvium, thus introducing an inversely acting regional driver. A process continuum of downstream (mass effect and species sorting due to solute characteristics) and lake effects (species sorting due to inverse solute structuring) are likely drivers of BCC. The “hijacking” of solute dependent processes acting on MF can also be seen at site SC, where SDM model components were longitudinally structured by (alluvial and surface) water flow (shared fractions with AEM, Figure 5H).

### Impact of processes on BCC and MF

Our results showed that spatial mechanisms linked to hydrology can explain small but significant amounts of variance in BCC and even more in MF. These mechanisms exhibit different degrees of importance depending on flow conditions and general floodplain morphology. Comte and Del Giorgio (2011) suggested microbial consortia functionally respond to environmental change, such as changes in flow, either by the existing phylotypes adjusting their activity at the single cell level or by more competitive phylotypes replacing non-adapted ones. In either pathway, a specific metabolic outcome can be achieved. Hence, an alternation between the two scenarios is dependent on initial community composition and the duration and strength of change in environmental variables. Lotic systems that show large amplitudes in physico-chemical changes in conjunction with physical stress are often dominated by specialists and high turnover (i.e., strong coupling of BCC and MF), whereas systems with smaller amplitudes in environmental change represent niches for more generalist species (Freimann et al., 2013a). Generalists may adjust functionality faster and more flexibly in response to environmental change as long as changes remain within the boundaries of their metabolic capabilities or they are not out-competed by specialists when a steady state persists over time (Freimann et al., 2013b).

We found that enzymes were structured either different (i.e., site RC, Figures 3A,D) or similar (i.e., site SC, Figures 4A,D) to bacteria assemblage structure. The processes working at site SC might be more stable over time, thus favoring a more specialized community composition (e.g., due to a constant interaction with the alluvium). Regional processes at site RC in October (i.e., AEM components and its shared fractions, Figure 5F) indicate initial reestablishment of asymmetric environmental conditions driven by hydrology. Here, MF reacted more quickly to changing nutritional states than BCC, indicating generalist prevalence. Generally, the differing importance of processes showed that BCC is less constrained by surface and hyporheic water flow compared to MF, indicating relatively large functional redundancy/plasticity within communities.

Alteration of hydrology due to changing climate conditions (e.g., reduced precipitation and/or extreme precipitation events) or altered flow regime due to hydropower production (which normally means a reduction in hydrologic connectivity) will likely be manifested as changes in BCC and MF (Singer et al., 2010). Contemporary communities and single-cell metabolic capabilities in conjunction with the degree and strength in fluctuation of environmental gradients (hydrologic and non-hydrologic related) will determine BCC on the long term and thus landscape-scale bacterial beta diversity (Freimann et al., 2013a). A permanent reduction in hydrological driven physico-chemical fluctuations will influence ecosystem functioning as spatially independent communities are likely to emerge, whereas spatially structured communities will be reduced (Singer et al., 2010). Under this scenario, it is unclear if bacteria would perform equally during connectivity pulses because microbial labor division along a flow path could be restricted by a loss of local plasticity previously provided by bacterial generalists.

This study sheds light on the importance of hydrologic connectivity on BCC and MF at the landscape scale. Future studies should establish which drivers connected with hydrological gradients ultimately lead to patterns in BCC and MF. Incorporating carbon and nutrient concentrations and qualities into developed models as well as *in situ* sediment characteristics also could better disentangle species sorting from mass and neutral effects. We note that BCC and MF were assessed for attached biofilms on glass beads. Measuring BCC in the pore water and of *in situ* sediment could provide additional information about bacterial mobility and habitat colonization dynamics. Together, this could help to develop holistic models able to quantify which combinations of community-based effects and degree of environmental fluctuations play major roles in forming BCC and MF at a given spot in the regional context of hydrologic linkages. Furthermore, a better understanding of bacterial community assembly, dynamics and function within floodplains can contribute to the development of optimal strategies in floodplain management with the aim of conserving or even improving ecosystem functioning.

## Author contributions

Conceived and designed the experiments: RF, HB, SF, and CR. Performed the experiments: RF, HB, and SF, CR. Analyzed the data: RF. Interpreted the data: RF, HB, SF, and CR. Contributed reagents/materials/analysis tools: RF, HB, SF, and CR. Wrote the paper: RF, HB, SF, and CR.

### Conflict of interest statement

The authors declare that the research was conducted in the absence of any commercial or financial relationships that could be construed as a potential conflict of interest.
