# Supplementary material for: Hydrologic linkages drive spatial structuring of bacterial assemblages and functioning in alpine floodplains
Source: Front Microbiol. 2015 Nov 3;6:1221. doi: 10.3389/fmicb.2015.01221 (PMC4630579; doi:10.3389/fmicb.2015.01221)
Supplement: Supplementary file 2 [file Image2.PDF]

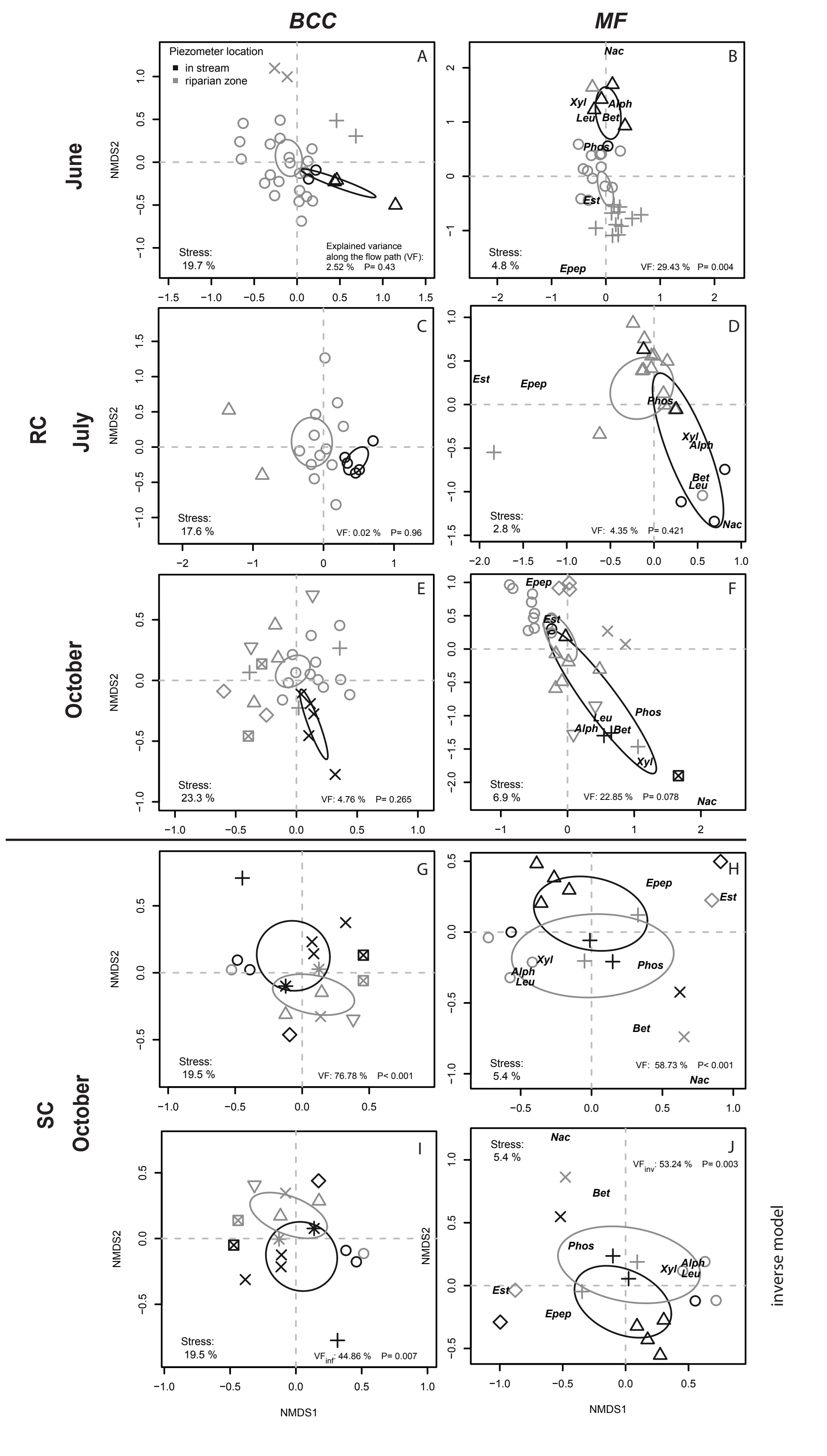

Figure S2

Non-metric multidimensional scaling (NMDS) to assess longitudinal (upstream-downstream) bacterial and functional gradient. Bray-Curtis dissimilarities of Wisconsin transformed community fingerprinting results and raw enzyme data are used for ordinations. The resulting plots are rotated so that the Euclidean distance along the flow path measured from the uppermost piezometer (i.e., RCP1 and SCP1 in Fig. 1) shows highest correlation with axis 1 of the NMDS plots. The fitting of these distance vectors (VF) after rotation is a measure of how bacterial structure or function are structured along the flow path without taking into account connectivity effects (i.e. unlike AEM or MEM models). The left panel shows BCC and the right panel MF. Locations of the piezometers are light grey for riparian zone and dark grey for in-stream piezometers. Dispersion ellipses for riparian versus in-stream location are shown in the same color scheme and depict the standard error of weighted average scores of piezometer location (confidence limit = 0.95). The vector fitting (VF and VFinv) for the rotated NMDS and the stress values are given for each NMDS. Symbols represent the relative affiliation of MF or BCC derived from the cluster analysis, i.e. same symbols indicate a high similarity for this parameter. A-B: site RC in June, C-D: site RC in July, E-F: site RC in October, G-H: site SC in October, I-J: site SC in October with inversely rotated NMDS. Annotations for MF are slightly moved to avoid superimpositions.
