# Supplementary material for: Hydrologic linkages drive spatial structuring of bacterial assemblages and functioning in alpine floodplains
Source: Front Microbiol. 2015 Nov 3;6:1221. doi: 10.3389/fmicb.2015.01221 (PMC4630579; doi:10.3389/fmicb.2015.01221)
Supplement: Supplementary file 1 [file Image1.PDF]

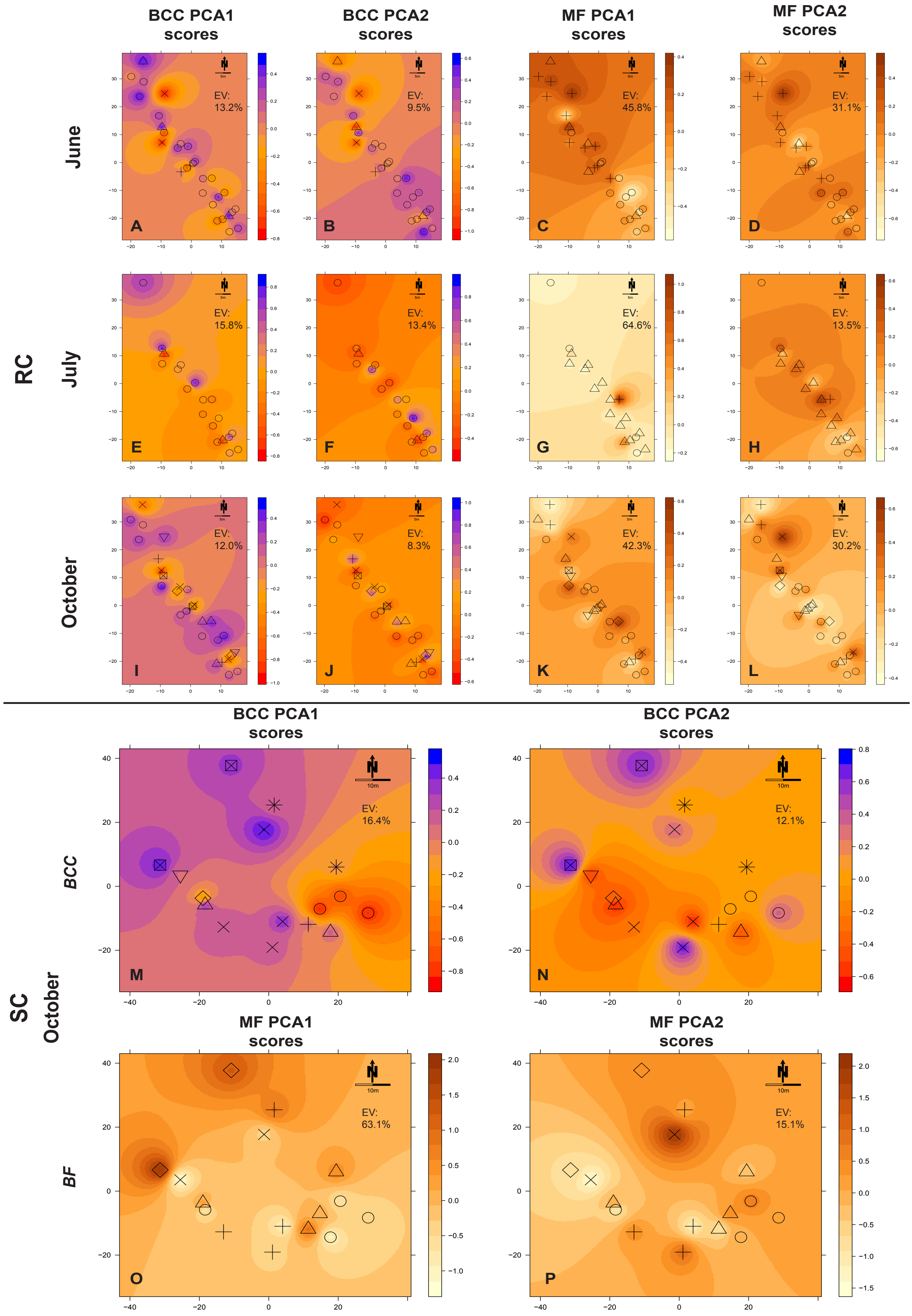

Figure S1

Spatial maps of inverse distant weight interpolations of PCA1 and PCA2 scores for BCC (pink-yellow color scheme) and MF (orange color scheme). The piezometer locations are depicted and symbols represent the relative affiliation of MF or BCC derived from the cluster analysis. Upper panel represents the RC floodplain during the different seasons. Lower panel represents the SC floodplain. EV: Explained Variation.
